# Impact of Galectin-Receptor Interactions on Liver Pathology During the Erythrocytic Stage of *Plasmodium berghei* Malaria

**DOI:** 10.3389/fimmu.2021.758052

**Published:** 2021-11-24

**Authors:** Yifan Wu, Shiguang Huang, Siyu Xiao, Jian He, Fangli Lu

**Affiliations:** ^1^ Department of Parasitology, Zhongshan School of Medicine, Sun Yat-sen University, Guangzhou, China; ^2^ School of Stomatology, Jinan University, Guangzhou, China; ^3^ Public Experimental Teaching Center, Sun Yat-sen University, Guangzhou, China; ^4^ Department of Clinical Laboratory, The Seventh Affiliated Hospital, Sun Yat-sen University, Shenzhen, China; ^5^ Key Laboratory of Tropical Disease Control of Ministry of Education, Sun Yat-sen University, Guangzhou, China

**Keywords:** *Plasmodium berghei*, liver, macrophage, Gal-9, Tim-3, IFNα, IFNγ, TREM-1

## Abstract

Hepatopathy is frequently observed in patients with severe malaria but its pathogenesis remains unclear. Galectins are evolutionarily conserved glycan-binding proteins with pleiotropic roles in innate and adaptive immune responses, and exhibit pivotal roles during *Plasmodium* spp. infection. Here, we analyzed the impact of blockage of galectin-receptor interactions by treatment with alpha (α)-lactose on liver immunopathology during the erythrocytic stage of malaria in mice infected with *Plasmodium berghei* ANKA (*Pb*ANKA). Our results found that compared with *Pb*ANKA-infected mice (malarial mice), blockage of galectin-receptor interactions led to decreased host survival rate and increased peripheral blood parasitemia; exacerbated liver pathology, increased numbers of CD68^+^ macrophages and apoptotic cells, and increased parasite burden in the livers on days 5 and 7 post infection (p.i.) as well as increased mRNA expression levels of galectin-9 (Gal-9) and its receptor, the T cell immunoglobulin domain and mucin domain protein 3 (Tim-3), interferon (IFN)α, IFNγ, and the triggering receptor expressed on myeloid cells (TREM)-1 in the livers or spleens of *Pb*ANKA-infected mice on day 7 p.i. Observed by transmission electron microscopy, the peritoneal macrophages isolated from malarial mice with α-lactose treatment had more pseudopodia than those from malarial mice. Measured by using quantitative real-time reverse transcription-polymerase chain reaction assay, the mRNA expression levels of Gal-9, IFNα, IFNβ, IFNγ, and TREM-1 were increased in the peritoneal macrophages isolated from malarial mice with α-lactose treatment in comparison of those from malarial mice. Furthermore, significant positive correlations existed between the mRNA levels of Gal-9 and Tim-3/IFNγ/TREM-1 in both the livers and the peritoneal macrophages, and between Gal-9 and Tim-3/TREM-1 in the spleens of malarial mice; significant positive correlations existed between the mRNA levels of Gal-9 and IFNγ in the livers and between Gal-9 and IFNα in the peritoneal macrophages from malarial mice treated with α-lactose. Our data suggest a potential role of galectin-receptor interactions in limiting liver inflammatory response and parasite proliferation by down-regulating the expressions of IFNα, IFNγ, and TREM-1 during *Pb*ANKA infection.

## Introduction

*Plasmodium falciparum* can cause severe disease manifested by cerebral malaria, difficulty breathing, difficulty urinating, hypoglycemia, lactic acidosis, severe anemia, jaundice, and liver involvement (1). In malaria patients with jaundice, histopathological changes of damaged hepatocytes, congestion of liver cells, hemozoin deposition, inflammatory infiltrates, and cholestasis as well as hyperplastic Kupffer cells have been demonstrated (2–4). Kupffer cell activation is an effective mechanism for regulating the host response to malaria liver-stage infection (5). In the erythrocytic stage of *P. falciparum* malaria, parasitized red blood cells (pRBCs) are sequestered within human small blood vessels. The degraded hemozoin (malarial pigment) is phagocytized by local tissue macrophages such as Kupffer cells and alveolar macrophages (6, 7). During *Plasmodium* liver-stage infection, Kupffer cells from *Plasmodium*-infected livers produce high levels of hepatocyte growth factor, which plays multiple roles during primary malaria hepatocyte infection and triggers apoptosis of infected hepatocytes (5). So far, several members of the triggering receptor expressed on myeloid cells (TREM) family including TREM-1, TREM-2, TREM-3, and TREM-4 have been identified, in which activation of TREM-1 amplifies inflammation, whereas TREM-2 signaling reduces inflammation (8–10). It has been reported that TREM-1 is a master regulator of Kupffer cell activation, which escalates chronic liver inflammatory responses, activates hepatic stellate cells, and promotes liver fibrosis (11). A study has demonstrated that *Trem-*2-deficient mice infected with *P. berghei* ANKA (*Pb*ANKA) sporozoites are more susceptible to liver-stage infection than their wild-type counterparts, and TREM-2 is involved in host responses against the malaria parasite (12).

Galectins are evolutionarily conserved glycan-binding proteins with diverse roles in innate and adaptive immune responses (13). Evidence showed that galectins are also involved in host–microbial recognition and pathogen subversion of immune responses (14). Galectin-9 (Gal-9) plays a critical role in regulating the fate of effector T cells, and can induce apoptosis of some T cell subsets by binding to the T cell immunoglobulin domain and mucin domain protein 3 (Tim-3) (13, 15). Gal-9 is highly expressed in the liver and has diverse roles in hepatic immune homeostasis and inflammation (16). A study found that Gal-9 is released during acute falciparum malaria infection from patients of Thailand, and the plasma levels of Gal-9 reflect malaria severity (17). The increase of Tim-3/Gal-9 expression levels may play an important role in the liver damage during *Pb*ANKA infection in mice (18). Kupffer cells are the predominant source of Gal-9 within the liver, and the circulating levels of Gal-9 in the plasma are elevated and Gal-9 expression is up-regulated in Kupffer cells in livers of chronic hepatitis C virus patients compared to normal controls (19).

Type I interferons (IFN-I) are a family of cytokines with a wide range of effects on innate and adaptive immune cells during infection with viruses, bacteria, parasites, and fungi (20). IFN-I-associated responses have been reported in malaria patients (21). IFN-I-signaling mediates an inflammatory reaction in the liver, which controls parasite numbers during *Plasmodium* liver-stage infection, and can be either detrimental or beneficial to the host depending on host-parasite genetics during blood-stage infection (22). *Plasmodium* infection also triggers an IFN-I response that mediates an inflammatory reaction in the liver against liver-stage malaria parasite (23, 24). In addition, activation of the IFN-I pathway may contribute to the pathogenesis of cerebral malaria (25). Blockage of Gal-9/Tim-3 pathway leads to increased IFN-I production in the lung of *Pb*ANKA-infected mice and murine peritoneal macrophages co-cultured with *Pb*ANKA-infected red blood cells *in vitro* (26). However, so far, the mechanisms of liver histopathology in the erythrocytic stage of malaria have not been systematically studied. Therefore, the present study was designed to define the relevance and role of galectins on macrophages in *Pb*ANKA-induced liver immunopathology in the erythrocytic stage. To analyze the role of galectin-receptor interactions in liver pathology during the erythrocytic stage of malaria, a murine model of *Pb*ANKA infection in the erythrocytic stage with or without blockage of galectin-receptor interactions were studied, here we identified a central role for galectin-receptor interactions in the survival rate, peripheral blood parasitemia/tissue parasite burden, liver pathology, and cytokine expressions in malarial mice. These findings provide the basis for developing galectin-receptor-targeted strategies to either prevent or attenuate malaria liver pathology.

## Materials and Methods

### Ethical Statement

*In vivo* experiments were approved by the Animal Experimentation Ethics Committee of Zhongshan School of Medicine on Laboratory Animal Care at Sun Yat-sen University (No. 2016-081) and were carried out in strict accordance with the Institutional Guidelines for Care and Use of Laboratory Animals.

### Mice, Treatment With α-Lactose, and Experimental Infections

Female Kunming mice (outbred, 6-8 weeks old) were obtained from the Animal Center of Sun Yat-sen University, and *Pb*ANKA parasites were used throughout the study. Some mice were injected intraperitoneally (i.p.) with 300 mM of α-lactose solution in phosphate buffered saline (PBS) twice daily starting from day 1 post infection (p.i.) until the day mice were sacrificed. Animals were sacrificed using CO_2_ asphyxiation and the appropriate organs were taken for further analysis 12 h after the last treatment (27). A total of 48 mice were used in the experiments: 16 mice were injected i.p. with 10^6^ *Pb*ANKA-pRBCs (e.g. malarial mice); 16 mice were injected i.p. with 10^6^ *Pb*ANKA-pRBCs and treated with α-lactose; 8 mice were injected i.p. with α-lactose alone, and 8 mice were injected with equal volume of PBS as negative controls. Mortality was monitored daily. Peripheral blood parasitemia was monitored daily by Giemsa-stained thin blood smears from the tail vein of mice. Erythrocyte counts were performed with a hematocytometer, and more than 1,000 RBCs were counted by light microscopy (×100) to determine the percentage of pRBCs. Eight *Pb*ANKA-infected mice were sacrificed for examination on days 5 and 7 p.i., respectively.

### Histopathology

For histopathological analysis, the livers from *Pb*ANKA-infected mice, *Pb*ANKA-infected mice with α-lactose treatment, or control mice were harvested and immediately fixed in 10% buffered formaldehyde (Guangzhou Chemical Reagent Factory, China) for 48 h. Five-micrometer-thick sections of the organs from mice were stained with hematoxylin and eosin (H&E) (Sigma-Aldrich, China) and evaluated for histological changes. A semi-quantitative score described previously was used to score liver inflammation and damage. Changes of the histopathological features in liver tissues were classified based on the severity of four histological criteria: architecture loss, sequestration of pRBCs in microvessels, pigment deposition, and inflammation (4, 28). The histopathological scores were graded on a scale of 0 to 3 [nil (0), partial loss (1), moderate loss (2), and total loss (3)]. The highest possible total score was 12 (4 histological criteria × 3 as highest scale). Score 0 means no histopathological change and score 12 refers to the most severe histopathological change (28). Overall liver tissues were analyzed at a magnification of × 100 under light microscopy by counting 10 fields of each section from each mouse in each group. All the analyses were performed by two researchers.

### Immunostaining for CD68 in the Livers and Morphometric Analysis

Immunohistochemistry was carried out using the streptavidin–biotin–peroxidase complex (SABC) method. Tissue sections (5-μm) were deparaffinized and rehydrated in distilled water. Heat-induced antigen retrieval was carried out in an 800-W microwave oven for 30 min. Endogenous peroxidase activity was blocked by incubation with 3% hydrogen peroxide in methanol for 10 min at 37°C. Nonspecific binding was blocked by incubation in 5% bovine serum albumin (Sigma-Aldrich) in PBS (pH 7.4) for 10 min at room temperature. The sections were incubated with rabbit anti-CD68 (Wuhan Boster Biological Engineering Co., Ltd., Wuhan, China) (1:200 dilutions) overnight at 4°C, and then with secondary antibodies. Sections incubated with secondary antibodies only were used as isotype controls. Signals were detected with a SABC kit and developed in diaminobenzidine tetrahydrochloride (Zhongshan Golden Bridge Technology, Beijing, China). The sections were counterstained with hematoxylin and examined under a light microscope. CD68^+^ macrophages were identified by dark-brown staining and were quantified using images captured with a digital camera system and analyzed by using Image-Pro Plus (Image Z1 software, Media Cybernetics, MD, USA). The number of cells in each field was determined under high power field as well as the area of each field (0.015066 mm^2^). The density of positive cells was expressed as the number of cells per square millimeter.

### Immunofluorescence Assay

For primary antibody incubation, liver tissues were incubated with mouse anti-mouse CD68 monoclonal antibody (IgG1; 1:200; Abcam, Cambridge, UK) and rabbit anti-Tim-3 polyclonal antibody (IgG1; 1:200 dilution; Abcam) or rabbit anti-Gal-9 monoclonal antibody (IgG1; 1:200 dilution; Bioss, Beijing, China) overnight at 4°C. Subsequently, PBS (pH 7.4) washed sections were incubated with anti-mouse IgG (H + L), F (ab’)_2_ fragment (Alexa Fluor. 488 Conjugate) (2 mg/ml, 1:200 dilution; Cell Signaling Technology, MD, USA) or anti-rabbit IgG (H + L), F (ab’)_2_ fragment (Alexa Fluor. 555) (2 mg/ml, 1:200 dilution; Cell Signaling Technology) for 1 h at room temperature in a dark chamber. After washing 3 times with PBS, the slides were mounted with polyvinylpyrrolidone antifade mounting medium (Beyotime, Haimen, China) with DAPI (1: 50,000; Sigma-Aldrich) in a dark chamber. For negative controls, a set of liver tissue slides was incubated under similar conditions without the primary antibodies. CD68^+^-macrophages were identified by their green fluorescence in liver, whereas Tim-3^+^ and Gal-9^+^ cells were identified by their red fluorescence, once CD68 and Gal-9 or CD68 and Tim-3 superimposed in one image that would appear as yellow fluorescence under a fluorescence microscope (Olympus BX63, Tokyo, Japan). Two researchers performed the observations.

### TUNEL Staining

Formalin-fixed paraffin embedded liver tissue sections (5-μm) were detected using TUNEL assay (Wuhan Boster Biological Engineering Co., Ltd.). Negative control was prepared by omission of terminal deoxynucleotidyl transferase. Apoptotic cells were determined under high power field as well as the area of each field (0.015066 mm^2^). The density of positive cells was expressed as the number of cells per square millimeter.

### Isolation of Murine Primary Peritoneal Macrophages

Female Kunming mice were used in this study. Naive mice, mice with α-lactose treatment, and *Pb*ANKA-infected mice with or without α-lactose treatment on day 5 p.i. were injected i.p. with 2 ml 3% thioglycollate broth (Sigma-Aldrich) solution in PBS once daily for 3 days. Animals were sacrificed and their peritoneal lavage fluid were spun at 800 g at 4°C for 5 min, and the pelleted peritoneal macrophages were re-suspended and seeded at 5 × 10^5^ cells/well in 12-well plates (Corning, NY, USA). After 4 h at 37°C in a 5% CO_2_ atmosphere, cells were washed and isolated. Samples were stored at −80°C until subjected to further analysis.

### Transmission Electron Microscopy

The peritoneal macrophages from *Pb*ANKA-infected mice, *Pb*ANKA-infected mice with α-lactose treatment, or control mice were isolated by centrifugation at 1 000 g for 3 min. The precipitates were immediately fixed in 3% glutaraldehyde and 1% osmium tetroxide (both in 100 mM PBS, pH 7.2) overnight before being dehydrated through a series of graded ethanol solutions. The fixed tissues were then embedded in SPIPon 812 Embedding Kit (Structure Probe Inc., West Chester, PA, USA) following the manufacture’s instruction. Ultrathin sections (70 nm) were cut from the embedded tissues using the Leica EM UC6 ultramicrotome (Leica Microsystems, Wetzlar, Germany) and mounted on formvar-coated grids. The sections were then stained for 15 min in aqueous 1% uranyl acetate followed by 0.2% lead citrate, and were then analyzed under a JEM100CX-II transmission electron microscope (JEOL Ltd., Tokyo, Japan) at an accelerating voltage of 100 kV.

### Measurement of mRNA Expression by Using Quantitative Real-Time Reverse Transcription-Polymerase Chain Reaction (qRT-PCR) Assay

Total RNA was extracted from about 100 mg of mouse liver and spleen tissues or peritoneal macrophages of each group using a commercial RNA Extraction Kit (TaKaRa Bio Inc., Shiga, Japan) according to the manufacturer’s protocol. The quality of total RNA was analyzed by running 5 μl of each RNA sample on a 1.0% agarose gel stained with ethidium bromide. The quantity of total RNA was estimated by measuring the ratio of absorbance at 260 and 280 nm using a NanoDrop ND-1000 spectrophotometer (NanoDrop Technologies, DE, USA). First-strand cDNA was constructed from 1.0 μg of total RNA with oligo (dT) as primers using a PrimeScript™ II 1st Strand cDNA Synthesis Kit (TaKaRa Bio Inc.) following the manufacturer’s protocol, and cDNA was stored at −80°C until use. To determine the mRNA levels of IFNα, IFNβ, IFNγ, TREM-1, TREM-2, Gal-9, and Tim-3 in tissues or macrophages of different groups of mice, qRT-PCR was performed using SYBR Green QPCR Master Mix (TaKaRa Bio Inc.) according to the manufacturer’s instructions. For liver parasite burden, mRNA level of *Pb*ANKA 18S rRNA measured by qRT-PCR is shown as -∆∆Ct values. Primers for the qRT-PCR are listed in **Table 1**. Briefly, a total of 10 μl reaction mixture contained 5.0 μl of SYBR^®^ Premix Ex TaqTM (2×), 0.5 μl of each primer (10 pM), 3.0 μl of dH_2_O, and 1.0 μl of cDNA (0.2 μg/μl). Amplification was pre-denaturized for 30 s at 95°C followed by 43 cycles of 5 s at 95°C and 20 s at 60°C with a LightCycler^®^ 480 instrument (Roche Diagnostics, AL, USA). Specific mRNA expression levels were normalized to that of the housekeeping gene, β-actin, and the results are expressed as fold change compared with uninfected controls (with or without α-lactose treatment).

**Table 1:** Primer sequences of genes used for quantitative real-time reverse transcription-polymerase chain reaction assay.

Genes	Forward primer (5′→3′)	Reverse primer (5′→3′)	Accession
β-actin	TGGAATCCTGTGGCATCCATGAAAC	TAAAACGCAGCTCAGTAACAGTCCG	NC_000071.6
IFNα	CTTTGGATTCCCGCAGGA	TGTAGGACAGGGATGGCTTGA	NC_000070.6
IFNβ	TGAATGGAAAGATCAACCTCACCTA	CTCTTCTGCATCTTCTCCGTCA	NC_000070.6
IFNγ	GGAACTGGCAAAAGGATGGTGAC	GCTGGACCTGTGGGTTGTTGAC	NC_000076.6
Gal-9	GTTGTCCGAAACACTCAGAT	ATATGATCCACACCGAGAAG	NC_000077.6
Tim-3	CCACGGAGAGAAATGGTTC	CATCAGCCCATGTGGAAAT	NC_000077.6
TREM-1	GACTGCTGTGCGTGTTCTTTG	GCCAAGCCTTCTGGCTGTT	NC_000083.6
TREM-2	CTGGAACCGTCACCATCACTC	CGAAACTCGATGACTCCTCGG	NC_000083.6
*Pb*ANKA 18S rRNA	AAGCATTAAATAAAGCGAATACATCCTTAC	GGAGATTGGTTTTGACGTTTATGTG	NW_672388.1

### Statistical Analysis

Results of experimental studies were reported as mean ± SD. Statistical analysis of the data was performed using Wilcoxon rank sum test, Student’s *t*-test, and one-way ANOVA followed by Bonferoni’s multiple comparison tests using SPSS software for windows (version 19.0; SPSS Inc., Chicago, IL, USA). All graphs were performed using GraphPad Prism software and a value of *P* < 0.05 was considered statistically significant.

## Results

### Blockage of Galectin-Receptor Interactions Decreased the Survival Rate and Increased Peripheral Blood Parasitemia/Tissue Parasite Burden in *Pb*ANKA-Infected Mice

**Figure 1:**
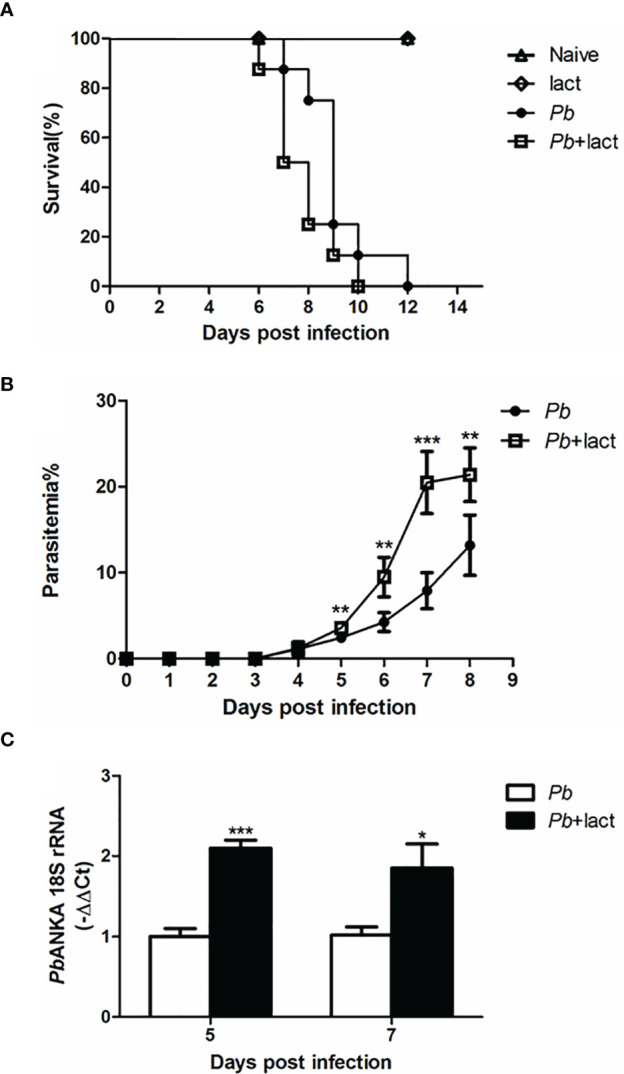
Survival rate, peripheral blood parasitemia, and liver parasite burden in *Pb*ANKA-infected mice with or without α-lactose treatment. **(A)** Survival rate. Malarial mice (n = 8) died between days 7 and 12 p.i. Malarial mice treated with α-lactose (n = 8) died between days 6 and 10 p.i. **(B)** Peripheral blood parasitemia levels of malarial mice and malarial mice treated with α-lactose were determined through blood smears at the indicated time points. Parasitemia are shown as mean ± SD. **P < 0.01, malarial mice treated with α-lactose on days 5, 6, and 8 p.i. *vs*. malarial mice on days 5, 6, and 8 p.i.; ***P < 0.001, malarial mice treated with α-lactose on day 7 p.i. *vs*. malarial mice on day 7 p.i. There were 4 mice per group, and data are representative of those from two experiments. **(C)** Parasite burden estimated using mRNA level of *Pb*ANKA 18S rRNA in the livers was measured by using qRT-PCR. Values are means from triplicate measurements, and data are shown as −ΔΔ Ct values. ***P < 0.001, malarial mice with α-lactose treatment on day 5 p.i. *vs*. malarial mice on day 5 p.i.; *P < 0.05, malarial mice with α-lactose treatment on day 7 p.i. *vs*. malarial mice on day 7 p.i. There were 4 mice per group, and data are representative of those from two experiments.

Malarial mice died between 7 and 12 days p.i., while malarial mice treated with α-lactose died between 6 and 10 days p.i. (**Figure 1A**). Malarial mice treated with α-lactose developed a significantly higher peripheral blood parasitemia on days 5 (*P* < 0.01), 6 (*P* < 0.01), 7 (*P* < 0.001), and 8 (*P* < 0.01) p.i. compared with those of malarial mice (**Figure 1B**). In addition, measured by using qRT-PCR, there were significantly increased parasite burdens (*Pb*ANKA 18S rRNA) in the livers of malarial mice treated with α-lactose compared with those of malarial mice on days 5 (*P* < 0.001) and 7 (*P* < 0.05) p.i. (**Figure 1C**). Thus, blockage of galectin-receptor interactions increased peripheral blood parasitemia and tissue parasite burden, and shortened the survival of mice after *Pb*ANKA infection.

### Blockage of Galectin-Receptor Interactions Increased Liver Damage of *Pb*ANKA-Infected Mice

**Figure 2:**
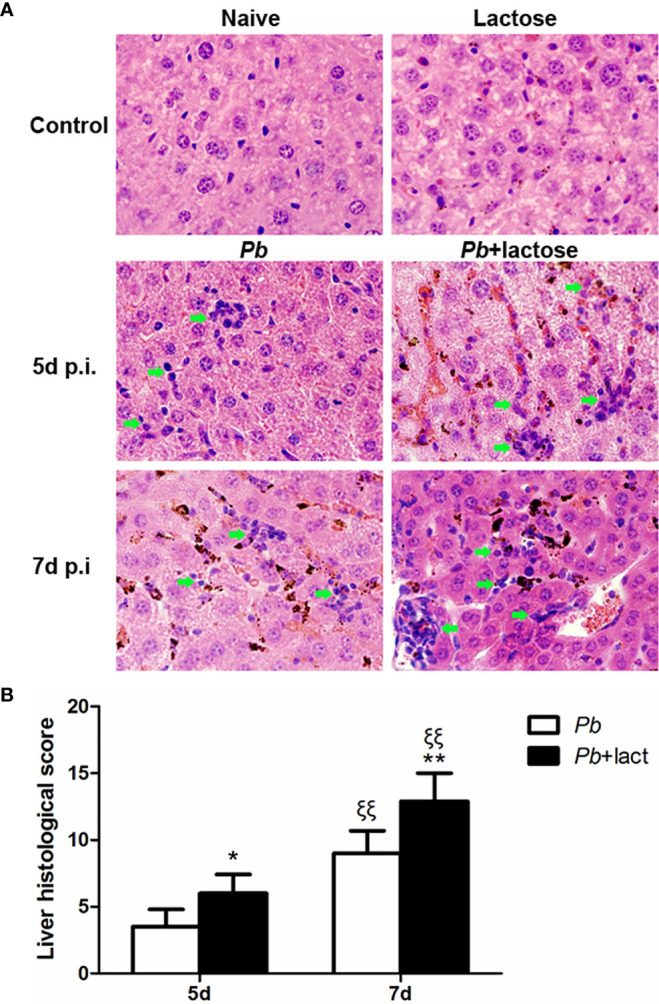
Histopathology of livers in *Pb*ANKA-infected mice with or without α-lactose treatment. **(A)** Histopathological changes in the livers. H&E stain. Inflammatory cell infiltrates were indicated with green arrow heads. **(B)** Histopathological score analysis of the livers. Data are represented as mean ± SD. Significant differences between groups were analyzed using Wilcoxon rank sum test. *P < 0.05 and ^ξξ^ *P* < 0.01, malarial mice with α-lactose treatment on day 5 p.i. and malarial mice on day 7 p.i. *vs*. malarial mice on day 5 p.i.; ^ξξ^ *P* < 0.01, malarial mice with α-lactose treatment on day 7 p.i. *vs*. malarial mice with α-lactose treatment on day 5 p.i.; **P < 0.01, malarial mice with α-lactose treatment on day 7 p.i. *vs*. malarial mice on day 7 p.i. There were 4 mice per group, and data are representative of those from two experiments.

Histopathological analysis showed no obvious morphological changes in the liver tissues of uninfected mice and uninfected mice treated with α-lactose. However, on days 5 and 7 p.i., obvious inflammation and necrotic lesions (indicated with green arrow heads) were observed in the livers of both malarial mice and malarial mice treated with α-lactose. Furthermore, extensive malarial pigment deposition was observed in the livers of malarial mice on day 5 p.i. and in the livers of malarial mice treated with α-lactose on days 5 and 7 p.i. (**Figure 2A**). Semi-quantitative inflammation scores based on pathological changes of the liver tissues showed that compared with malarial mice, significantly increased histopathological scores in the livers of malarial mice with α-lactose treatment on days 5 (*P* < 0.05) and 7 (*P* < 0.01) p.i. Compared with day 5 p.i., there were significantly increased histopathological scores in the livers of both malarial mice and malarial mice with α-lactose treatment on day 7 p.i. (*P <*0.01) (**Figure 2B**). The data suggested that more severe liver pathology developed in malarial mice with α-lactose treatment than that in malarial mice.

### Blockage of Galectin-Receptor Interactions Enhanced Apoptosis in the Livers of *Pb*ANKA-Infected Mice

**Figure 3:**
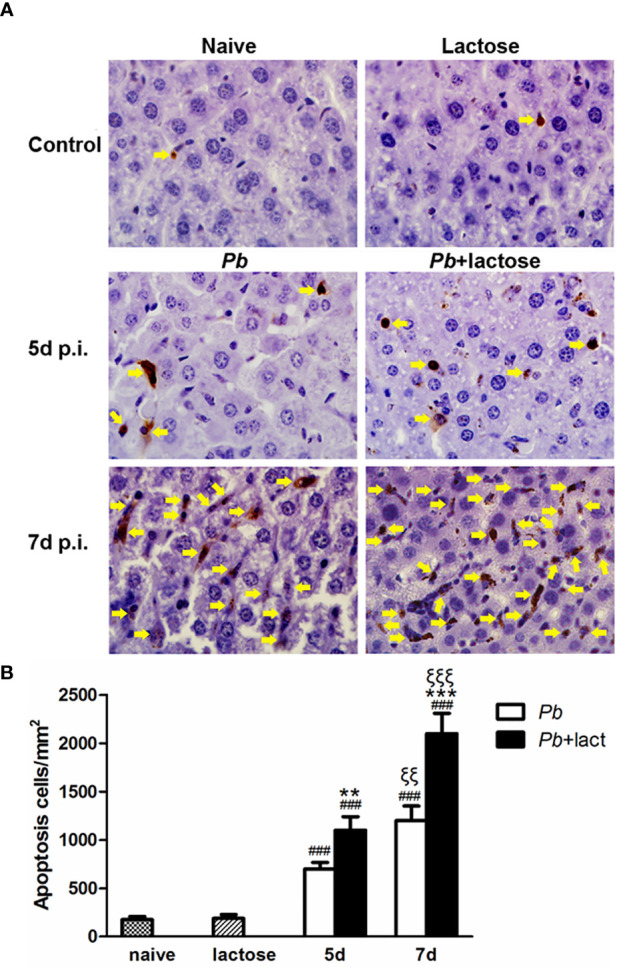
TUNEL staining of the liver tissues of *Pb*ANKA-infected mice with or without α-lactose treatment. Shown are representative liver sections by using TUNEL staining. **(A)** Apoptotic cells in the livers of naive mice, mice with α-lactose treatment, malarial mice, and malarial mice with α-lactose treatment on days 5 and 7 p.i. Apoptotic cells were indicated with yellow arrow heads. Original magnification × 1,000. **(B)** Morphometric analysis of apoptotic cells in the liver tissues. Shown are apoptotic cells per square millimeter. Data are presented as means ± SD; experiments were performed with 4 mice per group. ^###^ *P* < 0.001, malarial mice on day 5 p.i., malarial mice with α-lactose treatment on day 5 p.i., malarial mice on day 7 p.i., and malarial mice with α-lactose treatment on day 7 p.i. *vs*. naive group; **P < 0.01, malarial mice with α-lactose treatment on day 5 p.i. *vs*. malarial mice on day 5 p.i.; ^ξξξ^ *P* < 0.001, malarial mice with α-lactose treatment on day 7 p.i. *vs*. malarial mice with α-lactose treatment on day 5 p.i.; ***P < 0.001, malarial mice with α-lactose treatment on day 7 p.i. *vs*. malarial mice on day 7 p.i. ^ξξ^ *P* < 0.01, malarial mice on day 7 p.i. *vs*. malarial mice on day 5 p.i.

Apoptosis in the livers was measured by TUNEL staining. There were only a few apoptotic cells observed in the liver tissues of uninfected controls. On days 5 and 7 p.i., a great number of apoptotic cells were observed in the livers of both malarial mice and malarial mice treated with α-lactose. However, more apoptotic cells, scattered throughout the liver tissue section, were observed in the livers of malarial mice treated with α-lactose in comparison of those of malarial mice on days 5 and 7 p.i. (**Figure 3A**). Quantitative analysis of TUNEL positive cells showed that compared with uninfected controls, the numbers of apoptotic cells were significantly increased in the livers of malarial mice with or without α-lactose treatment on days 5 and 7 p.i. (*P* < 0.001). Compared with malarial mice, the numbers of apoptotic cells were significantly increased in the livers of malarial mice treated with α-lactose on days 5 (*P* < 0.01) and 7 (*P* < 0.001) p.i. (**Figure 3B**). Phagocytosis of pRBCs causes induction of apoptosis in macrophages through release of cytosolic factors from *P. falciparum* 3D7-infected mouse macrophages (29). The data demonstrated that blockage of galectin-receptor interactions may increase cellular apoptosis in the liver during the erythrocytic stage of malaria.

### Blockage of Galectin-Receptor Interactions Increased CD68^+^ Macrophages in the Livers of *Pb*ANKA-Infected Mice

**Figure 4:**
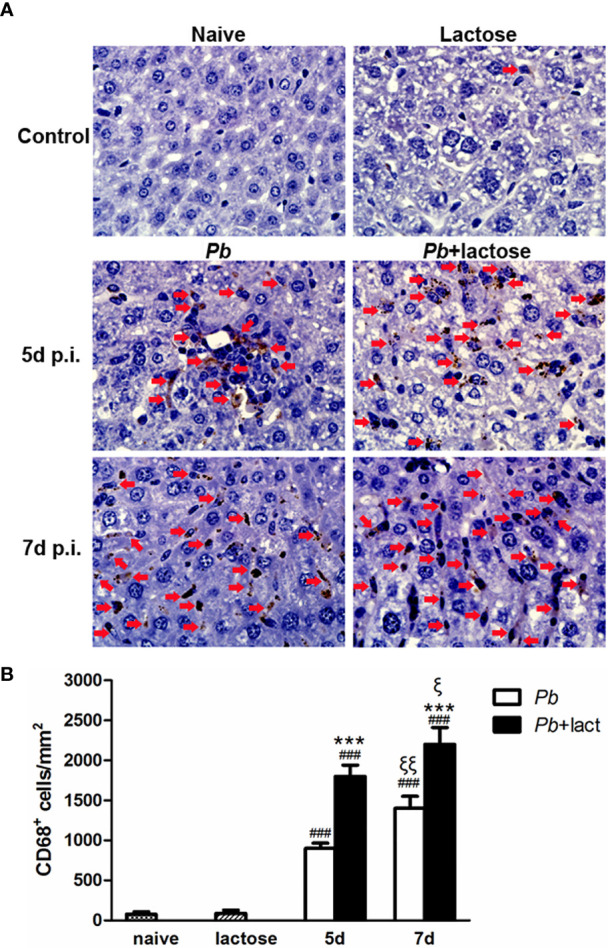
Immunohistochemical staining for CD68^+^ macrophages in the livers of *Pb*ANKA-infected mice with or without α-lactose treatment. **(A)** Immunohistochemical staining of CD68^+^ macrophages in the livers of uninfected mice, uninfected mice with α-lactose treatment, malarial mice, and malarial mice with α-lactose treatment on day 7 p.i. Positive cells were indicated with red arrow heads. Original magnification × 1,000. **(B)** Morphometric analysis of liver tissues. Shown are CD68^+^ macrophages per square millimeter. Data are presented as means ± SD; experiments were performed with three mice per group. ^###^ *P* < 0.001, malarial mice on day 5 p.i., malarial mice with α-lactose treatment on day 5 p.i., malarial mice on day 7 p.i., and malarial mice with α-lactose treatment on day 7 p.i. *vs*. naive group; ***P < 0.001, malarial mice with α-lactose treatment on day 5 p.i. *vs*. malarial mice on day 5 p.i.; ***P < 0.001, malarial mice with α-lactose treatment on day 7 p.i. *vs*. malarial mice on day 7 p.i.; ^ξ^ *P* < 0.05, malarial mice with α-lactose treatment on day 7 p.i. *vs*. malarial mice with α-lactose treatment on day 5 p.i.; ^ξξ^ *P* < 0.01, malarial mice on day 7 p.i. *vs*. malarial mice on day 5 p.i.

A few CD68^+^ macrophages (brown color) were observed in the liver tissues of uninfected controls. However, a large number of CD68^+^ macrophages were dispersed in the liver sections of malarial mice or malarial mice treated with α-lactose on days 5 and 7 p.i. (**Figure 4A**). Quantitative analysis of CD68^+^ macrophages in the liver tissues showed that compared with uninfected controls, there were significantly higher numbers of CD68^+^ macrophages in the livers of malarial mice on days 5 and 7 p.i. (*P* < 0.001). Compared with malarial mice, there were significantly increased CD68^+^ macrophages in the livers of malarial mice with α-lactose treatment on days 5 and 7 p.i. (*P* < 0.001). Compared with malarial mice or malarial mice with α-lactose treatment on day 5 p.i., there were significantly increased numbers of CD68^+^ macrophages in the livers of malarial mice and malarial mice with α-lactose treatment on day 7 p.i. (*P* < 0.01 and *P* < 0.05, respectively) (**Figure 4B**). It has been reported that the number of Kupffer cells is significantly increased and their phagocytic activity is enhanced during the erythrocytic stage of malaria in Sprague-Dawley rats infected with *Pb*ANKA (30). In addition, Kupffer cells show marked hypertrophy and hyperplasia and are filled with malarial pigment, and total Kupffer cell phagocytic activity in the liver during the erythrocytic phase of malaria is also markedly increased in Wistar rats infected with *Pb*ANKA (31). Our data indicate that blockage of galectin-receptor interactions may increase the activity of liver CD68^+^ macrophages in the erythrocytic stage of malaria, which is in agreement with the increased parasitemia and tissue parasite burden in malarial mice with α-lactose treatment.

### Gal-9 and Tim-3 Expressions on CD68^+^ Macrophages in the Livers After *Pb*ANKA Infection

**Figure 5:**
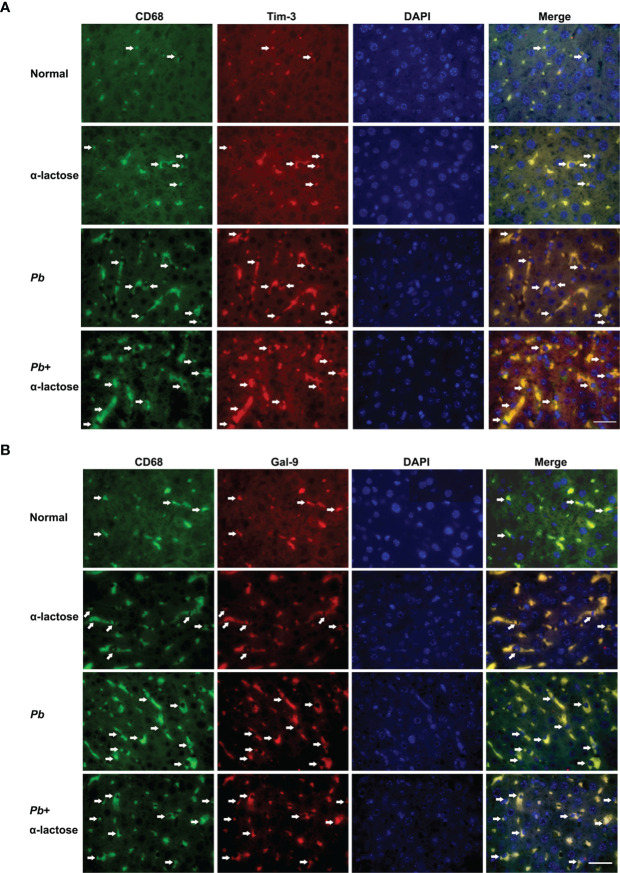
Immunofluorescence staining for CD68^+^-Gal-9^+^ and CD68^+^-Tim-3^+^ macrophages in the liver tissues of *Pb*ANKA-infected mice with or without α-lactose treatment. Double immunofluorescence showed expression of Gal-9 or Tim-3 on CD68^+^ macrophages. **(A)** CD68^+^-Gal-9^+^ macrophages in the liver tissues of malarial mice and malarial mice with α-lactose treatment on day 7 p.i. **(B)** CD68^+^-Tim-3^+^ macrophages in the liver tissues of malarial mice and malarial mice with α-lactose treatment on day 7 p.i. in comparison of naive and α-lactose-control. Positive cells were indicated with white arrow heads. Original magnification × 1,000.

Immunofluorescence assay showed that both Gal-9 (**Figure 5A**) and Tim-3 (**Figure 5B**) were expressed on the CD68^+^ macrophages in the liver tissues of all the groups; more CD68^+^-Gal-9^+^ and CD68^+^-Tim-3^+^ macrophages were observed in malarial mice and malarial mice treated with α-lactose in comparison of uninfected controls. The data suggested that CD68^+^-Gal-9^+^ and CD68^+^-Tim-3^+^ macrophages play a role in the inflammatory response in liver during the erythrocytic stage of malaria.

### Blockage of Galectin-Receptor Interactions Enhanced Gal-9, Tim-3, IFNα, IFNγ, and TREM-1 Expressions in the Livers or Spleens of *Pb*ANKA-Infected Mice

**Figure 6:**
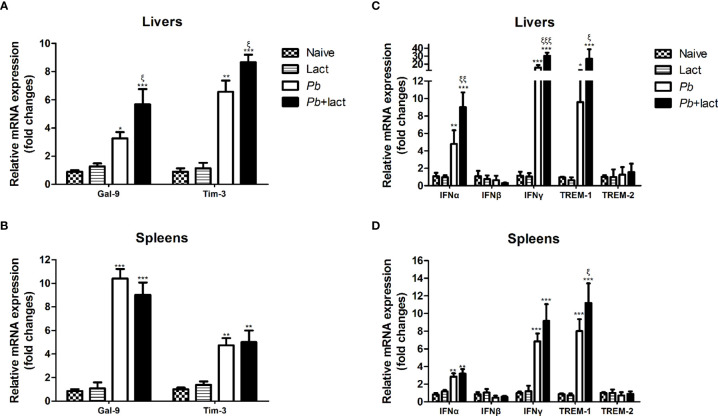
Relative mRNA expressions of Gal-9, Tim-3, IFNα, IFNβ, IFNγ, TREM-1, and TREM-2 in the liver **(A, C)** and spleen **(B, D)** tissues of *Pb*ANKA-infected mice with or without α-lactose treatment were detected by using qRT-PCR. Values are means from triplicate measurements, and data are presented as means ± SD; two independent experiments were performed with 4 mice per group. *P < 0.05, **P < 0.01, and ***P < 0.001, α-lactose-control, malarial mice on day 7 p.i., and malarial mice with α-lactose treatment on day 7 p.i. *vs*. naive mice; ^ξ^ *P* < 0.05, ^ξξ^ *P* < 0.01, and ^ξξξ^ *P* < 0.001, malarial mice with α-lactose treatment on day 7 p.i. *vs*. malarial mice on day 7 p.i.

Here, the mRNA expression levels of Gal-9, Tim-3, IFNα, IFNβ, IFNγ, TREM-1, and TREM-2 in the livers and spleens of *Pb*ANKA-infected mice with or without α-lactose treatment on day 7 p.i. were examined (**Figure 6**). Compared with uninfected controls, there were significantly increased Gal-9 (*P* < 0.05 and *P* < 0.001, respectively) and Tim-3 (*P* < 0.01 and *P* < 0.001, respectively) in the livers, and significantly increased Gal-9 (*P* < 0.001) and Tim-3 (*P* < 0.01) in the spleens of malarial mice and malarial mice with α-lactose treatment on day 7 p.i. Compared with malarial mice, there were significantly increased mRNA levels of Gal-9 and Tim-3 in the livers of malarial mice with α-lactose treatment on day 7 p.i. (*P* < 0.01).

Compared with uninfected controls, there were significantly increased mRNA expression levels of IFNα (*P* < 0.01 and *P* < 0.001, respectively), IFNγ (*P* < 0.001), and TREM-1 (*P* < 0.05 and *P* < 0.001, respectively) in the livers, and significantly increased levels of IFNα (*P* < 0.01), IFNγ (*P* < 0.001), and TREM-1 (*P* < 0.001) in the spleens of malarial mice and malarial mice with α-lactose treatment on day 7 p.i. Compared with malarial mice, there were significantly increased levels of IFNα (*P* < 0.01), IFNγ (*P* < 0.001), and TREM-1 (*P* < 0.05) in the livers and significantly increased TREM-1 level (*P* < 0.05) in the spleens of malarial mice with α-lactose treatment on day 7 p.i.

### Ultrastructural Observation of Peritoneal Macrophages by Transmission Electron Microscopy

**Figure 7:**
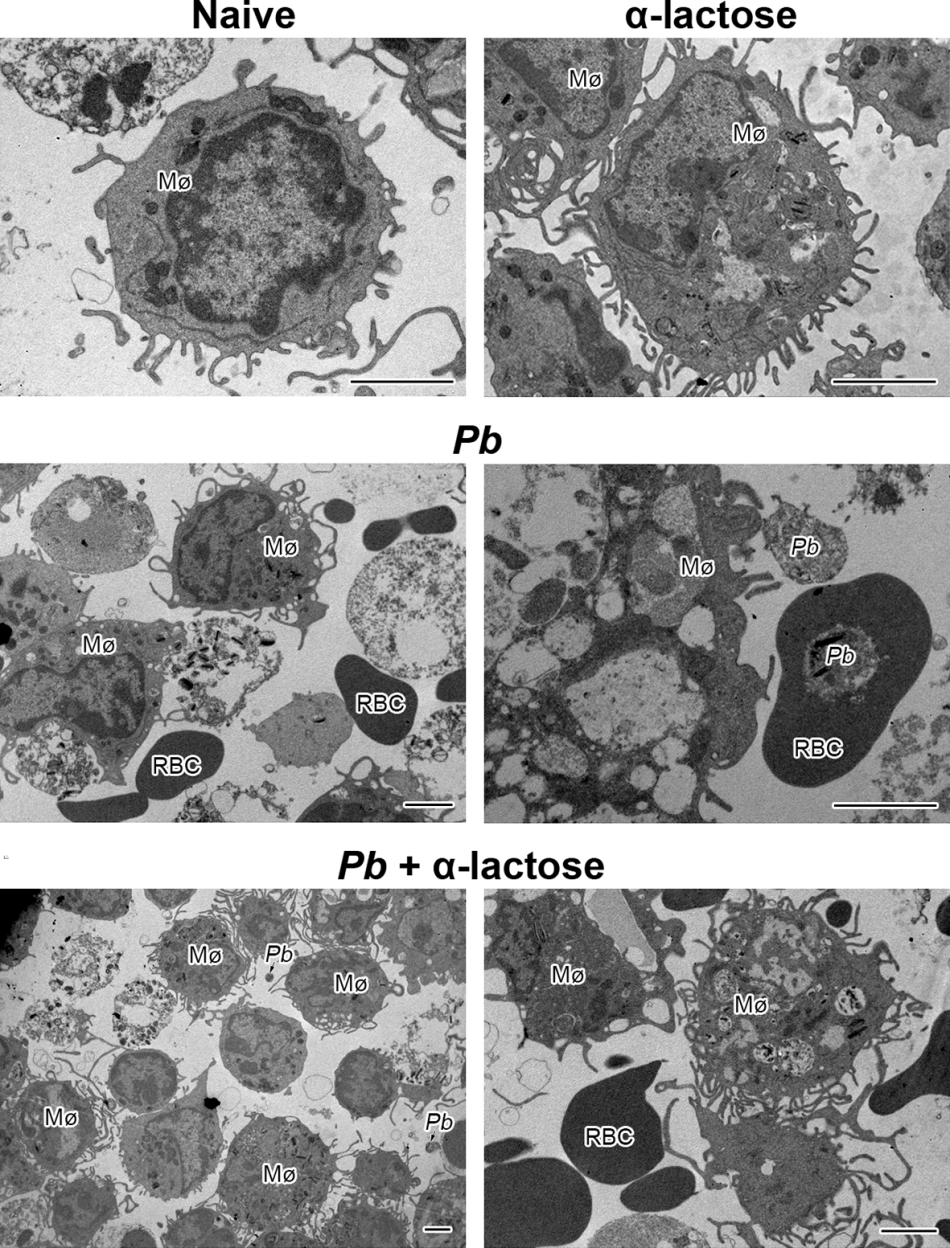
The ultrastructures of peritoneal macrophages from *Pb*ANKA-infected mice with or without α-lactose treatment. Mø, peritoneal macrophage; RBC, red blood cell; *Pb*, *P. berghei*. Bar = 2 μm.

The ultrastructures of peritoneal macrophages isolated from different groups of mice were observed by transmission electron microscopy, the peritoneal macrophages from *Pb*ANKA-infected mice with α-lactose treatment showed more pseudopodia in comparison of those from *Pb*ANKA-infected mice (**Figure 7**)

### Blockage of Galectin-Receptor Interactions Increased Gal-9, Tim-3, IFNα, IFNβ, IFNγ, and TREM-1 Expressions in the Peritoneal Macrophages Isolated From *Pb*ANKA-Infected Mice

**Figure 8:**
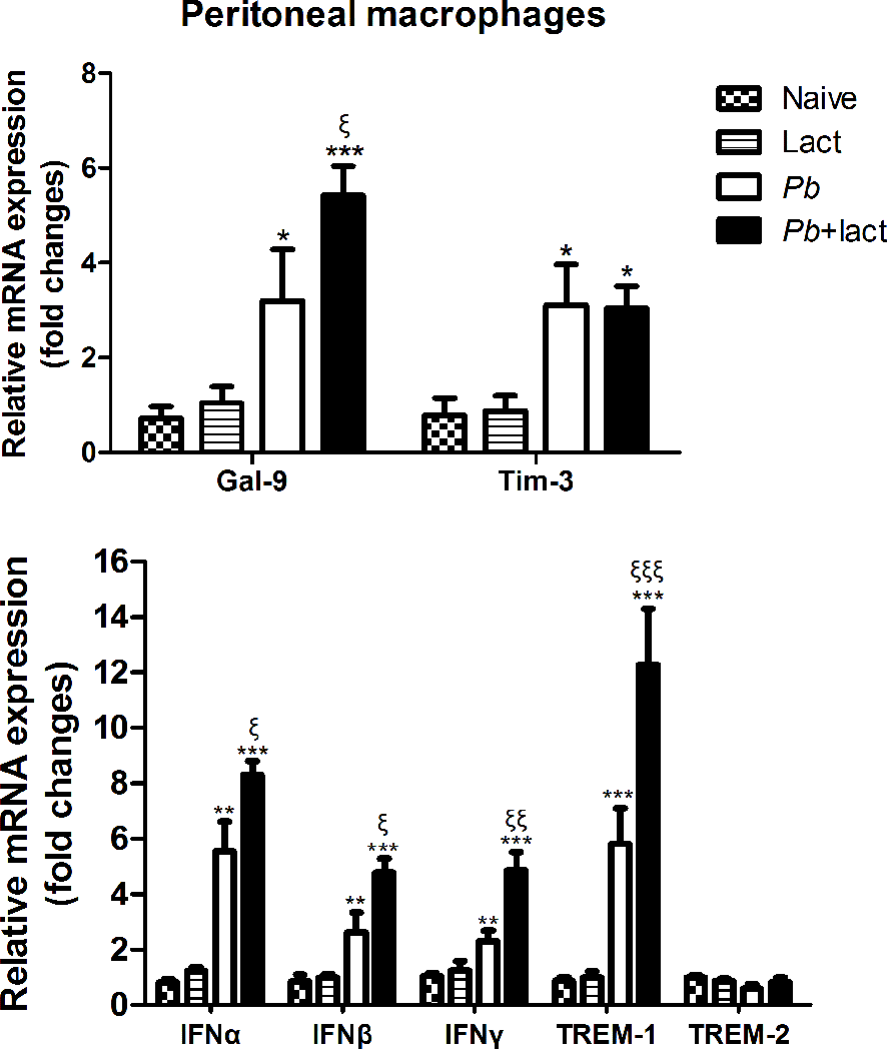
Relative mRNA expressions of Gal-9, Tim-3, IFNα, IFNβ, IFNγ, TREM-1, and TREM-2 expressions in murine peritoneal macrophages were examined *ex vivo* from *Pb*ANKA-infected mice with or without α-lactose treatment on day 7 p.i. by using qRT-PCR. Values are means from triplicate measurements, and data are presented as means ± SD; two independent experiments were performed. *P < 0.05, **P < 0.01, and ***P < 0.001, α-lactose-control, malarial mice on day 7 p.i., and malarial mice with α-lactose treatment on day 7 p.i. *vs*. naive group; ^ξ^ *P* < 0.05, ^ξξ^ *P* < 0.01, and ^ξξξ^ *P* < 0.001, malarial mice with α-lactose treatment on day 7 p.i. *vs*. malarial mice on day 7 p.i.

To test whether galectin-receptor interactions have an impact on functional activation of macrophages by *Pb*ANKA infection, we examined the mRNA expression levels of Gal-9, Tim-3, IFNα, IFNβ, IFNγ, TREM-1, and TREM-2 in the peritoneal macrophages *ex vivo* isolated from *Pb*ANKA-infected mice with or without α-lactose treatment on day 7 p.i. Compared with uninfected controls, there were significantly increased levels of Gal-9 (*P* < 0.05 and *P* < 0.001, respectively), Tim-3 (*P* < 0.05), IFNα (*P* < 0.01 and *P* < 0.001, respectively), IFNβ (*P* < 0.01 and *P* < 0.001, respectively), IFNγ (*P* < 0.01 and *P* < 0.001, respectively), and TREM-1 (*P* < 0.001) in the peritoneal macrophages isolated from both *Pb*ANKA-infected mice and *Pb*ANKA-infected mice treated with α-lactose. Compared with *Pb*ANKA-infected mice, there were significantly increased levels of Gal-9 (*P* < 0.05), IFNα (*P* < 0.05), IFNβ (*P* < 0.05), IFNγ (*P* < 0.01), and TREM-1 (*P* < 0.001) in those from *Pb*ANKA-infected mice treated with α-lactose (**Figure 8**). Studies has revealed a substantial deleterious role for IFN-I-signaling in mediating severe and lethal disease during *Pb*ANKA infection (22). IFN-γ is a key contributor to the pathogenesis of cerebral malaria in the *Pb*ANKA mouse model (32). Using *Pb*ANKA-infected ICR mice to investigate the involvement of TREM-1 during malaria infection, and found that mice with low parasitemia levels have low concentrations of plasma TREM-1 and vice versa. Therefore, TREM-1 plays important pro-inflammatory roles during malaria infection and may be one of the key mediators of the disease’s severity (33). The data suggested that blockage of galectin-receptor interactions induces the expressions of IFN-I, IFNγ, and TREM-1 genes, which may exacerbate liver damage of malarial mice.

### Correlations Between Gal-9/Tim-3 and IFNα, IFNβ, IFNγ, or TREM-1 Expression Levels *In Vivo* and *Ex Vivo*

**Figure 9:**
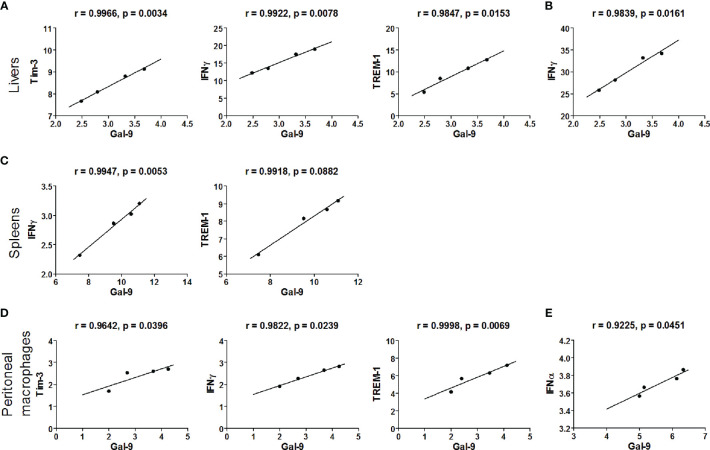
Correlation analysis between Gal-9 and Tim-3, IFNα, IFNβ, IFNγ, or TREM-1 mRNA expression levels in the livers, spleens, and the peritoneal macrophages *ex vivo* from malarial mice and malarial mice with α-lactose treatment (*n* = 4). Significant correlations between Gal-9 and Tim-3/IFNγ/TREM-1 level in the livers of malarial mice **(A)** and significant correlations between Gal-9 and IFNγ level in the livers of malarial mice with α-lactose treatment **(B)**. Significant correlations between Gal-9 and IFNγ/TREM-1 level in the spleens of malarial mice **(C)**. Significant correlations between Gal-9 and Tim-3/IFNγ/TREM-1 level in the peritoneal macrophages isolated from malarial mice **(D)** and significant correlations between Gal-9 and IFNα level in the peritoneal macrophages isolated from malarial mice with α-lactose treatment **(E)**. The *r* value generates for the theoretical line of best fit, and the *P* value indicates the significance of the correlation.

The correlations between the mRNA levels of Gal-9 and Tim-3/IFNα/IFNβ/IFNγ/TREM-1 in the livers, spleens, and peritoneal macrophages of *Pb*ANKA-infected mice or *Pb*ANKA-infected mice treated with α-lactose were evaluated, and only significant correlations were presented. There were significant correlations between the mRNA levels of Gal-9 and Tim-3 (r = 0.9966, *P* = 0.0034), Gal-9 and IFNγ (r = 0.9922, *P* = 0.0078), and Gal-9 and TREM-1 (r = 0.9847, *P* = 0.0153) in the livers of *Pb*ANKA-infected mice (**Figure 9A**), and there was significant correlation between the mRNA levels of Gal-9 and IFNγ (r = 0.9839, *P* = 0.0161) in the livers of *Pb*ANKA-infected mice treated with α-lactose (**Figure 9B**). There were significant correlations between the mRNA levels of Gal-9 and IFNγ (r = 0.9947, *P* = 0.0053), and Gal-9 and TREM-1 (r = 0.9918, *P* = 0.0882) in the spleens of *Pb*ANKA-infected mice (**Figure 9C**). There were significant correlations between the mRNA levels of Gal-9 and Tim-3 (r = 0.9642, *P* = 0.0396), Gal-9 and IFNγ (r = 0.9822, *P* = 0.0239), and Gal-9 and TREM-1 (r = 0.9998, *P* = 0.0069) in the peritoneal macrophages isolated from *Pb*ANKA-infected mice (**Figure 9D**), and there was significant correlation between the mRNA levels of Gal-9 and IFNα (r = 0.9225, *P* = 0.0451) in the peritoneal macrophages isolated from *Pb*ANKA-infected mice treated with α-lactose (**Figure 9E**). Taken together, significant positive correlations existed between the mRNA levels of Gal-9 and Tim-3/IFNγ/TREM-1 in both the livers and peritoneal macrophages, and between Gal-9 and Tim-3/TREM-1 in the spleens of *Pb*ANKA-infected mice; significant positive correlations existed between the mRNA levels of Gal-9 and IFNγ in the livers and between Gal-9 and IFNα in the peritoneal macrophages from *Pb*ANKA-infected mice treated with α-lactose. The data suggested that Gal-9/Tim-3 pathway may play an important role in the regulation of liver inflammatory response including upregulation of TREM-1, IFNγ, and IFNα expressions during the erythrocytic stage of *Pb*ANKA infection.

## Discussion

Hepatic dysfunction is one of the clinical features in severe falciparum malaria and a significant cause of morbidity and mortality among humans (4). However, the immunoregulatory mechanisms of hepatic injury during malaria are still poorly understood. To analyze the impact of galectin-receptor interactions on malaria-induced liver pathology, we employed *Pb*ANKA infection in a murine model. Our data showed that mice infected with *Pb*ANKA plus α-lactose treatment had lower survival rate, increased peripheral blood parasitemia and parasite burdens in the liver, more severe hepatocyte damage, and increased apoptotic cells in the liver compared with those of malarial mice. Moreover, our data found that the expression levels of Gal-9, Tim-3, IFNα, IFNβ, IFNγ, and TREM-1 were more upregulated in the livers, spleens, and peritoneal macrophages of malarial mice with α–lactose treatment relative to those of malarial mice without α-lactose treatment.

It has been documented that the liver serves as an effector against blood-stage malaria (34), wherein the liver endothelial system eliminates the parasitized erythrocytes possibly by phagocytosis (35). Macrophage phagocytosis is the first line of defense of the innate immune system against malaria parasite infection (36). Hepatic histological analysis in parasitemic cases of Malawian children with fatal encephalopathy caused by *P. falciparum* revealed that increased numbers of hemozoin-laden Kupffer cells were invariably present with a strong association with histological evidence of cerebral malaria (4). Kupffer cell hyperplasia, malarial pigment within the Kupffer cells, and liver cell necrosis with portal inflammation, steatosis, and cholestasis were observed in the livers of Indian patients with fatal malaria (6). Apoptosis of Kupffer cells and portal tract lymphocytes is a significant finding in the livers of severe *P. falciparum* malaria cases (4). In this study, immunohistochemical analysis revealed that more infiltration of CD68^+^ macrophages were observed in the liver tissues of malarial mice treated with α-lactose in comparison of malarial mice on days 5 and 7 p.i., indicating blockage of galectin-receptor interactions may increase the activity of macrophages during the erythrocytic stage of malaria.

Galectins, a conserved family of immunomodulatory animal lectins, are widely expressed in different tissues and a number of immune cell populations (37), and are involved in inflammation, immune responses, cell migration, autophagy, and signaling (38). So far 15 galectins have been identified in mammals (39), in which Gal-9 is expressed in a variety of tissues such as small intestine, thymus, kidney, spleen, lung, cardiac and skeletal muscles, reticulocyte, and brain, and is particularly abundant in the liver (40). It has been reported that blocking the Tim-3/Gal-9 pathway results in the increase of IFNγ production from hepatic effector T cells, which lead to exacerbated local inflammation and liver damage in a mouse model of concanavalin A-induced hepatitis, indicating the important role of Tim-3/Gal-9 signaling in the maintenance of hepatic homeostasis (41). Kupffer cells are the resident macrophage population localized within the liver sinusoid and serve as gatekeepers (42). Hepatic macrophages can arise from proliferating resident macrophages and circulating monocytes that originate from the bone marrow, which are also recruited to the injured liver (43, 44). Our previous study found blocking interactions of Gal-9 and its receptors (Tim-3, CD44, CD137, and protein disulfide isomerase) using α-lactose enhances inflammatory response and exacerbates malaria-associated acute lung injury and tissue damage, indicating that Gal-9 interaction with its receptors plays a role in the development of malaria-associated acute lung injury in *Pb*ANKA-infected mouse model (26). In the present study, our data showed that by immunofluorescence assay, increased CD68^+^-Gal-9^+^ and CD68^+^-Tim-3^+^ Kupffer cells/macrophages were observed in the liver tissues of malarial mice with or without α–lactose treatment on day 7 p.i., indicating that the activity of Kupffer cells was enhanced during the erythrocytic stage of malaria, which were consistent with that of the increased mRNA levels of Gal-9 and Tim-3 in the livers and peritoneal macrophages of malarial mice and malarial mice treated with α-lactose. It has been reported that Gal-9 production by Kupffer cells is related to the innate and adaptive immune response (19). Our data indicate that Gal-9/Tim-3 signaling may be involved in inflammatory immune responses to *Pb*ANKA infection in liver damage during the erythrocytic stage of malaria, suggesting an important mechanism for severe malaria.

Kupffer cells and infiltrating macrophages/monocytes are the major innate immune cells in the liver (45). Kupffer cells filled with damaged and parasitized erythrocytes and hemoglobin degradation pigment were observed in mice infected with *P. chabaudi* by electron microscopy (46). To further elucidate the role and regulation of galectins in activated macrophages during malaria, we investigated whether blockage of galectin-receptor interactions by α-lactose contribute to macrophage activation during *Pb*ANKA infection. Direct *ex vivo* examination of macrophages revealed that the peritoneal macrophages of *Pb*ANKA-infected mice with α-lactose treatment had more pseudopodia in comparison of those of malarial mice. In addition, the peritoneal macrophages from *Pb*ANKA-infected mice with α-lactose treatment were able to express higher levels of Gal-9, IFNα, IFNβ, IFNγ, and TREM-1 in comparison of those from malarial mice, suggesting that galectin-receptor interactions play a role in regulating macrophage inflammatory response during the erythrocytic stage of malaria. Our data indicate that Kupffer cells/macrophages are the predominant source of Gal-9, Tim-3, IFNα, IFNβ, IFNγ, and TREM-1, and play an important role in the liver damage during the erythrocytic stage of malaria induced by *Pb*ANKA infection.

IFN-I signaling is critical for innate immune elimination of liver-stage of *P. yoelii* parasite infection (24). It has been reported that the absence of functional IFNα/β receptor pathway and IFNγ pathway reduces murine experimental cerebral malaria-associated brain pathology induced by blood-stage *Pb*ANKA infection (47). IFNα and IFNβ can induce excessive inflammation (48). Activation of the IFN-I pathway may contribute to pathogenesis of cerebral malaria in Malawi (25). IFNγ plays a critical role in mediating protective immunity against both the pre-erythrocytic and blood-stage malaria parasites (49). In the present study, our data showed significantly increased levels of IFNα and IFNγ in both the livers and spleens of malarial mice treated with α-lactose in comparison of malarial mice on day 7 p.i. It has been reported that IFN-I signaling, suppresses Th1 responses and causes experimental severe and fatal disease during *Pb*ANKA infection (50). IFN-I‐signaling during the blood‐stage of infection appears to promote CD8^+^ T‐cell responses in humans and mice, and may induce CTL‐mediated immune‐pathology (22). Anti-Gal-9 Ab treatment has been shown to significantly elevate IFNγ production of murine splenocytes (51). Our data further demonstrated the involvement of IFNα and IFNγ genes in inflammation of liver injury during the erythrocytic stage of malaria.

Apoptosis plays a principal role in normal tissue development and in the pathogenesis of different diseases (52). Gal-9 combines with Tim-3 to induce apoptosis in Th1 cells (15). Apoptosis in macrophages is responsible for immune-depression and pathological effects during malaria (29). IFNγ limits splenic T cell numbers during *Pb*ANKA infection by promoting apoptosis (53). The number of apoptotic cells in the spleen were increased during acute *P. chabaudi* infection and involved both T cells, B cells, and macrophages (54). In the current study, the malarial mice with α-lactose treatment displayed significantly higher number of TUNEL-positive cells in the livers compared with those of malarial mice, suggesting that blockage of galectin-receptor interactions may alter *Pb*ANKA-induced inflammatory immune responses by regulating IFN-I and Type II IFN production in malarial mice.

TREM family receptors play important roles in the regulation of both innate and adaptive immune response (55), and TREM genes are expressed mainly in cells of monocyte/macrophage lineage (56). TREM-1 plays an important role in the amplification of inflammation (57). Higher soluble form of TREM-1 levels were observed among children in Ghana suffering from severe malaria compared with those of uncomplicated malaria, indicating that high plasma levels of TREM-1 were associated with the development of severe malaria (58). TREM-1 has been implicated as a biomarker of macrophage activation in human malaria patients (59). TREM-2 signaling is initially associated with negative regulation of macrophage activation (60). In this study, our data showed that the expression of TREM-1 was significantly increased in the livers, spleens, and peritoneal macrophages of malarial mice treated with α-lactose, suggesting that the hepatic injury induced by *Pb*ANKA is caused by the local production of cytokines that activates inflammatory cells in the liver. It has been reported that the increase of plasma Gal-9 levels during falciparum malaria infection is capable of identifying severe cases and tracking the inflammation process (17). In the present study, our data indicate that Gal-9/Tim-3 pathway may regulate liver inflammation through increasing the expressions of TRRM-1, IFNγ, and IFNα in *Pb*ANKA-infected mice. However, the mechanism of how the IFN-I and TREM-1 influence liver injury during the erythrocytic stage of malaria required further investigation.

In conclusion, we assessed the involvement of galectins in liver inflammatory responses to murine malaria induced by *Pb*ANKA. Our data demonstrated that, blocking galectin-receptor interactions exhibited increased liver histopathology and increased activity of Kupffer cells in the livers of malarial mice due to an increase in Kupffer cell numbers, which was associated with the upregulated levels of Gal-9, Tim-3, IFNα, IFNγ, and TREM-1 in the erythrocytic stage of malaria. Our findings reveal an important role of galectin-receptor interactions in regulating the expressions of IFNα, IFNβ, IFNγ, and TREM-1 genes during *Plasmodium* parasite infection and provide new insight into innate immunity to the protozoan parasites. Further studies will be necessary to elucidate how galectin-receptor interactions modulate the functions of Kupffer cells/macrophages in malaria-associated liver damage.

## Data Availability Statement

The original contributions presented in the study are included in the article/supplementary material. Further inquiries can be directed to the corresponding authors.

## Ethics Statement

The animal study was reviewed and approved by The Animal Experimentation Ethics Committee of Zhongshan School of Medicine on Laboratory Animal Care at Sun Yat-sen University (No. 2016-081).

## Author Contributions

FL designed the experiments, analyzed data, wrote, and edited the manuscript. SH revised and edited the manuscript. YW, SX, and JH conducted experiments and analyzed data. All authors contributed to the article and approved the submitted version.

## Funding

This work was supported by the National Natural Science Foundation of China (81971955); the Natural Science Foundation of Guangdong Province (2019A1515011667, 2021A1515012115); 2021 Graduate Education Innovation Plan Project of Guangdong Province (2021SFKC003); Guangdong Basic and Applied Basic Research Foundation (2019A1515011318); Shenzhen Science and Technology Innovation Commission Basic Research Foundation (JCYJ20180307150637015); the Undergraduate Teaching Quality Engineering Project of Sun Yat-sen University (SYSU Undergraduate Education [2021]93, SYSU Undergraduate Education [2020]72); the Open Project of Key Laboratory of Tropical Disease Control of Ministry of Education, Sun Yat-sen University (2020ZX02).

## Conflict of Interest

The authors declare that the research was conducted in the absence of any commercial or financial relationships that could be construed as a potential conflict of interest.

## Publisher’s Note

All claims expressed in this article are solely those of the authors and do not necessarily represent those of their affiliated organizations, or those of the publisher, the editors and the reviewers. Any product that may be evaluated in this article, or claim that may be made by its manufacturer, is not guaranteed or endorsed by the publisher.
